# Acute *Brucella* infection associated with splenic infarction: a case report and review of the literature

**DOI:** 10.3389/fcimb.2023.1234447

**Published:** 2023-10-04

**Authors:** Limei Shi, Shuang Wang, Xiaohua Li, Xin Li, Yuxiang Li, Yang Wang

**Affiliations:** Center of Infectious Disease and Pathogen Biology, Department of Infectious Diseases, The First Hospital of Jilin University, Changchun, China

**Keywords:** brucellosis, *Brucella abortus*, splenic infarction, vasculitis, acute pancreatitis

## Abstract

*Brucella* infection often involves multiple organ systems with non-specific clinical manifestations, and cutaneous involvement is uncommon. Splenic infarction and leukocytoclastic vasculitis also rarely occur together in the course of brucellosis infection. We report the case of a 47-year-old man with *Brucella* combined with splenic infarction. The patient presented with fever; large liver, spleen, and lymph nodes; muscle and joint pain; positive laboratory tests for blood cultures (*Brucella abortus*); and imaging suggestive of splenic infarction. After treatment with streptomycin, doxycycline, and rifampicin, the patient’s clinical symptoms and splenic damage improved. Detailed history taking, correct interpretation of laboratory results, and knowledge of rare complications of human brucellosis facilitate early diagnosis and treatment of the disease.

## Introduction

Brucellosis is one of the most common zoonotic diseases caused by infection with the bacterial genus *Brucella*. Human brucellosis is distributed globally and is prevalent in developing countries. Approximately 500,000 new cases of human brucellosis are reported worldwide each year (Al Jindan, 2021). Approximately 40,000 new cases are diagnosed annually in China, and it remains a serious public health problem (Tao et al., 2021). The species *Brucella melitensis* (which infects goats and sheep) and *Brucella abortus* (cattle) cause significant economic losses for animal husbandry and severe human disease (Gwida et al., 2010). The infection is transmitted to humans primarily through the consumption of unpasteurized dairy products and undercooked meat or through direct contact with infected animals, the placenta, or aborted fetuses (D'anastasio et al., 2011). The disease has a strong occupational profile. Among the occupations that come into direct contact with animals and their products, the most affected are those of rural workers and butchers. The clinical manifestations of the disease are often non-specific, and the most typical signs of infection are fever; malaise; excessive sweating; muscle and joint pain; weakness; and enlargement of the liver, spleen, and lymph nodes (Kose et al., 2014; Edathodu et al., 2021). It can affect all body systems with complications such as osteoarthritis, hepatitis, central nervous system dysfunction, cardiovascular disease, respiratory manifestations, orchitis or epididymitis, and hemophagocytic syndrome. Because many cases remain unrecognized due to atypical clinical presentations, inaccurate diagnosis, and inadequate surveillance, the case numbers should only be considered a minimal estimate (Yagupsky et al., 2019). Splenic infarction is very rare in human brucellosis patients. Here, we report a case of acute brucellosis combined with splenic infarction and elevated tumor marker and amylase levels.

## Case report

A 47-year-old man living in the countryside with a history of exposure to cattle was admitted to our hospital with a 2-week history of abdominal distension and a 9-day history of fever. Initially, the patient also presented with fatigue, myalgia, and joint pain. Within 2 weeks, he lost 7.5 kg of body weight. His temperature reached 38.9°C for 9 days. Before he came to our hospital, he was treated in a local hospital for 9 days. His local test results showed that his blood culture was negative, and abdominal computed tomography (CT) showed splenic infarction and portal vein enlargement. The *Brucella* serum tube agglutination test (SAT) was negative (1:25). His symptoms had no improvement with 6-day ceftriaxone [2.0, quaque die, intra-veineuse drip (QD, iv. D)] treatment. Then, he was discharged from the local hospital and visited our hospital.

At admission, his physical examination results were as follows: blood pressure, 132/72 mmHg; high-grade fever, 39.0°C; tachycardia, 102 beats/min; submandibular, cervical, and axillary enlarged lymph nodes were 10–20 mm, soft, freely moveable, and nontender; he had hepatomegaly, with the liver felt 2.0 cm below the ribs; and splenomegaly, with the spleen felt 5.0 cm below the ribs.

**Figure 1:**
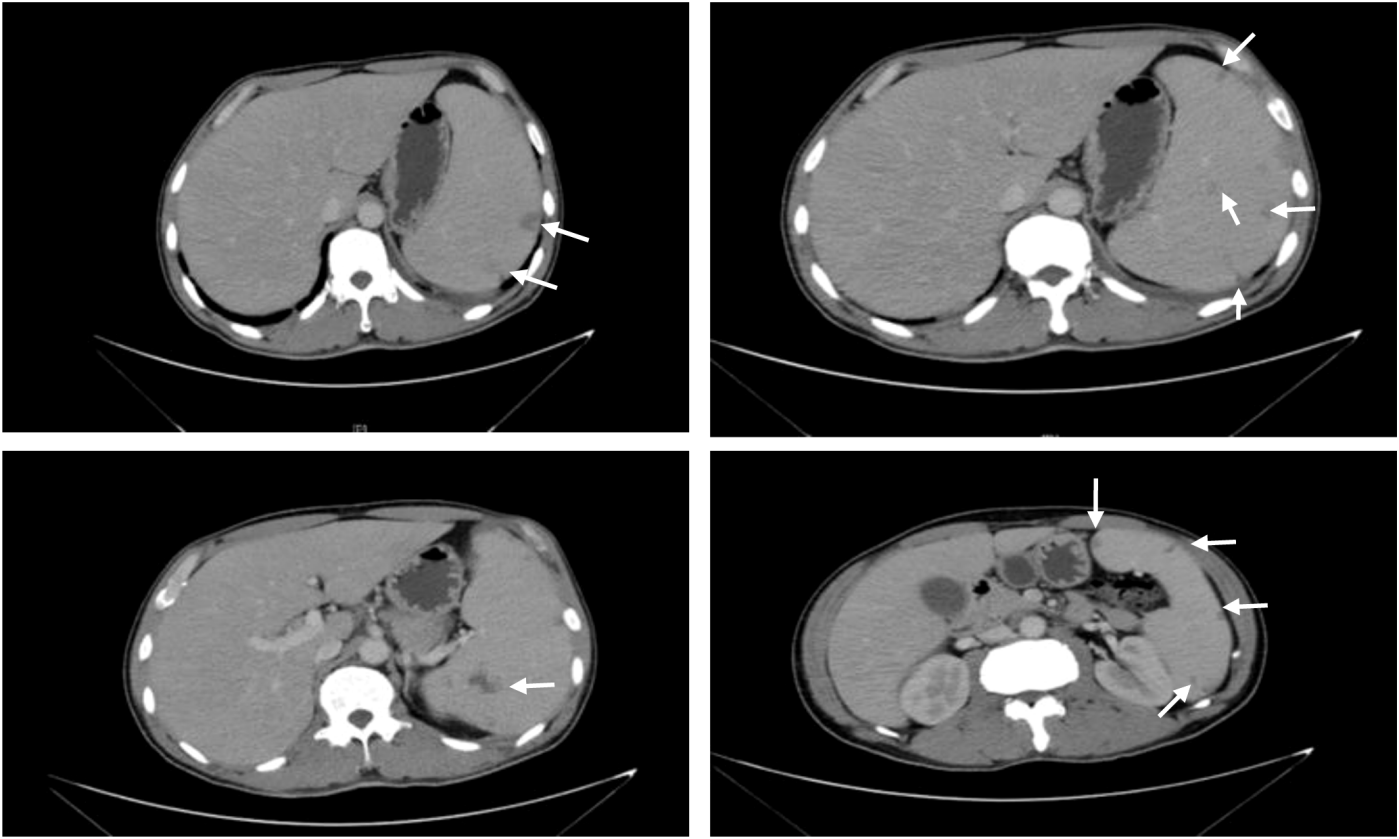
Abdominal CT scan. CT plain scan and tertiary enhancement of the abdomen before treatment: splenomegaly with multiple patches of slightly hypointense opacity within, CT value approximately 32 HU, considering the possibility of infarction. The white arrows indicate the hypointense shadow of the spleen.

The laboratory results were as follows: white blood cell counts of 3.2 × 10^9^/L (normal range: 3.5–9.5) and hemoglobin of 100 (normal range: 130–175) g/L. Liver enzymes and cardiac enzymes were increased: aspartate aminotransferase of 329.9 (normal range: 15–40) U/L, alanine aminotransferase of 217.1 (normal range: 9–50) U/L, alkaline phosphatase of 417.4 (normal range: 45–125) U/L, and lactate dehydrogenase of 284 (normal range: 120–250) U/L. Pancreatic enzymes were increased: amylase of 151.8 (normal range: 35–135) U/L and lipase of 127.8 (normal range: 0–79) U/L. The tumor marker carbohydrate antigen (CA) 19-9 level was obviously increased at 240.1 (normal range: 0–37) U/mL. *Brucella* serum tests were sent again, and the results were as follows: STA was weakly positive (1:100), and Brucellosis antibody IgG was positive. Inflammatory marker levels were increased: C-reactive protein of 17.0 (normal range: 0–10) mg/L, erythrocyte sedimentation rate of 72 (normal range: 0–15) mm/h, and ferritin of 897.3 (normal range: 20–300) μg/L. Abdominal ultrasound showed hepatomegaly, splenomegaly, and multiple low-density lesions in the spleen. Enhanced abdominal CT showed hepatomegaly, splenomegaly, multiple low-density lesions in the spleen (prone to splenic infarction), portal vein enlargement, and a small amount of effusion in the pelvis (**Figure 1**). *Brucella abortus* was cultured and isolated from blood after 5 days.

**Figure 2:**
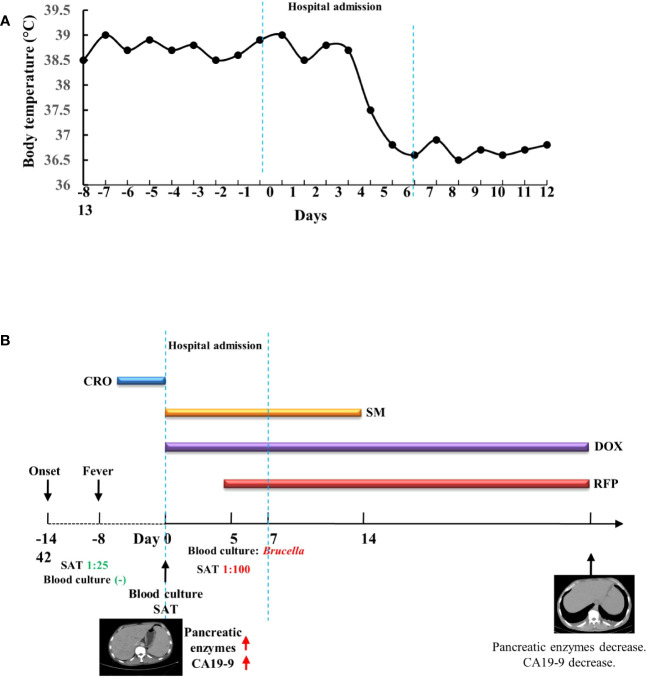
Timeline of the patient’s clinical presentation, relevant tests, and treatment. **(A)** Timeline of the patient’s body temperature. **(B)** Timeline of the patient’s tests and treatment. CRO, ceftriaxone; SM, streptomycin; DOX, doxycycline; RFP, rifampicin; SAT, test tube agglutination test.

**Figure 3:**
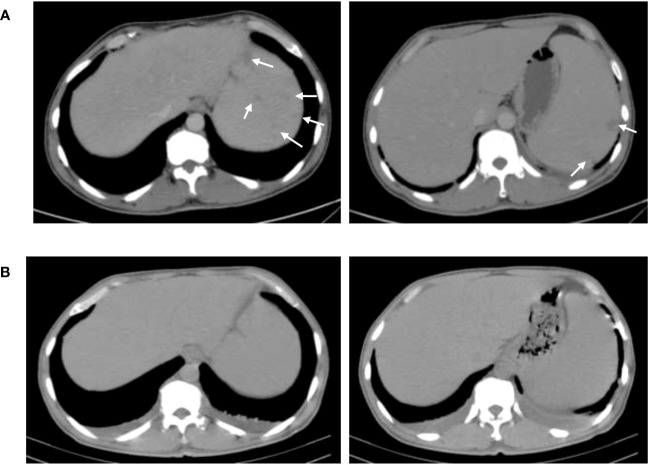
Comparison of abdominal CT results before and after treatment. **(A)** CT of the abdomen before treatment. **(B)** CT of the abdomen after 6-week treatment. The spleen has much fewer hypodense lesions than the former.

Because of the concerns about his long-term exposure to cattle, treatment after admission was started and consisted of doxycycline [0.1, bis in die, per os (BID, p.o.)] and streptomycin [15 mg/kg, quaque die, intramuscular injection (QD, IM)]. After verifying *Brucella abortus* infection by blood culture, rifampicin (0.6, QD, p.o.) was added to the combination therapy (**Figure 2**). The body temperature of the patient recovered to the normal level after anti-brucellosis treatment for 6 days. After 8 days of treatment, blood tests and abdominal ultrasound were performed. The blood test results were as follows: CA 19-9 decreased to 70.1 U/L. Abdominal ultrasound showed hepatomegaly, splenomegaly, and no low-density lesions in the spleen. The subsequent treatment with streptomycin was administered for 2 weeks, and the other two medicines were taken for 6 weeks. After 1 month of treatment, pancreatic enzymes levels and liver enzymes recovered to normal levels: the amylase 66 U/L, the lipase 55.2 U/L, aspartate aminotransferase 20 U/L, alanine aminotransferase 18 U/L, and alkaline phosphatase 88 U/L. After 6 weeks of treatment, CA 19-9 recovered to normal levels. Abdominal CT showed that the low-density lesions in the spleen were much less than the former (**Figure 3**).

## Discussion

With the rapid development of animal husbandry in China, the incidence of brucellosis has increased significantly in recent years. In 2021, the incidence rate of human brucellosis is 4.95/100,000 in China. It is difficult to diagnose because of its non-specific manifestations, and it is very important to take a detailed history and to differentiate it from other infectious diseases (D'anastasio et al., 2011). Lack of appropriate treatment in the acute phase may lead to localization of the bacteria in various tissues and organs and result in treatment failure, relapse, chronic course, focal complications, and high morbidity and mortality rates. Although humans are occasionally infected, the number of infections continues to be high each year, and the current vaccines are still flawed (de Figueiredo et al., 2015). Because *Brucella* grows slower than common bacteria, the results of routine blood cultures are often negative, which is also a reason for the high rate of missed diagnosis. Lower SAT titers cannot be used as a basis for excluding the diagnosis of human brucellosis, especially for patients during the first stage of the infection (Orduña et al., 2000). Moreover, elevated SAT titers are also very meaningful for the diagnosis of human brucellosis. Although the patient had two negative blood cultures and a low SAT titer, extended blood cultures and another SAT were performed. The bacteria were isolated and identified after 5 days, and the SAT titer increased four-fold. Detailed history taking, extended blood culture, and appropriate evaluation of SAT results are important for the diagnosis of human brucellosis.

Splenic infarction is common in the following conditions: thromboembolism, cardiovascular etiology or hypercoagulable state, acute infections causing rapid enlargement of the spleen, hematologic diseases, pulmonary embolism causes, and vasculitis (Antopolsky et al., 2009). Splenic infarction can be associated with a variety of infectious diseases, with intracellular microbial infections being the most common (Im et al., 2020). Splenic infarction is a rare complication of brucellosis, and only eight cases have been reported (**Table 1**). Among these cases, five patients had positive blood culture results. Left upper abdominal pain was common, although some patients did not have abdominal pain. The courses of antibiotic combination therapy were more than 6 weeks. After 2 to 4 months, the imaging tests returned to normal. The pathogenesis of splenic infarction in brucellosis patients is not yet clear. *Brucella* infection can lead to vasculitis (Odeh et al., 2000). In severe cases, visceral arteries may also be involved. Among the previously reported eight patients with brucellosis complicated with splenic infarction. There were only three cases combined with vasculitis, two of which were confirmed by histological examination in two cases (Uçmak et al., 2014; Wang et al., 2017; Saad et al., 2021). The reason for the splenic infarction in our patient was probably due to the rapid enlargement of the spleen, or it could have been caused by vasculitis. Because there is no pathological examination, the cause of the splenic infarction is unknown. Therefore, we need to add *Brucella* infection to the differentiation of vasculitis and splenic infarction as well (Saad et al., 2021).

**Table 1:** ** **A review of the literature on case reports of brucellosis complicated with splenic infarction.

Case	Career	Gender	Age	Clinical picture	Brucella species	Outcome	References
1	Unemployed	Male	17	Fever vomiting left abdominal tenderness weight loss of 9 kg	Not identified	Three months later, the splenic infarction improved.	(Alyousef et al., 2015)
2	Shepherd	Male	21	Fever left hypochondrium pain and splenomegaly	Culture negative	At 1 year of follow-up, no splenic infarction.	(Hachfi et al., 2012)
3	Farmer	Female	48	Fever, fatigue, chills, and abdominal pain	Not identified	Twelve weeks later, the splenic infarction disappeared.	(Alkan et al., 2022)
4	Animal husbandry	Male	39	Febrile, malignant, and painful	*Brucella mellitensis*	Six months later, his agglutination titers had decreased to 1/80 and no splenic lesions.	(Salgado et al., 2002)
5	Animal husbandry	Female	36	Fever, chills, weight loss, nausea, vomiting, and arthralgia	*Brucella mellitensis*	Two months later, the spleen was in normal view on magnetic resonance imaging.	(Dursun et al., 2015)
6	Not available	Male	17	Fever, malaise, loss of appetite, pain in the knee joints, abdominal pain (epigastric and in the abdominal left upper quadrant), and rash on legs	Not identified	No fever after 5 days of antibiotic treatment, the rash disappeared after 8 days, treatment was completed within 6 weeks, and there were no complications at the fourth month of follow-up.	(Uçmak et al., 2014)
7	Animal husbandry	Male	45	Fever, chills, fatigue, headache, arthralgias in both knees, intermittent pain in the left upper abdomen, and weight loss of 7 kg during the past 2 months	No culture	After 2 months, the complete blood cell count and the liver function test results are also normalized. Serum agglutination count decreased, and the spleen volume reduced.	(Lee et al., 2010)
8	Unemployed	Male	15	Fever and intermittent, diffuse abdominal pain, and weight loss of 5 kg	Culture negative	After 3 weeks of treatment, he had no fever or abdominal pain. After another 7 weeks, follow-up CT and ultrasound (US) examinations showed remarkable improvements in the bowel.	(Wang et al., 2017)

Cases of Brucella infection combined with acute pancreatitis have been reported, and this complication is not common in brucellosis patients (Suvak et al., 2016). In our case, the clinical manifestations of acute pancreatitis in this patient were not obvious. He had elevated levels of amylase, lipase, and the tumor marker CA 19-9. In the early stage, abdominal CT suggested a splenic infarction without imaging manifestations of pancreatic inflammation. CA 19-9 is a tumor marker closely related to pancreatic cancer, but it can also be elevated during pancreatic inflammation. The changes in pancreatic enzymes and the increase in tumor markers related to pancreatic cancer indicate that this patient had pancreatic damage. These tests returned to normal after standard combination therapy with doxycycline, rifampicin, and streptomycin.

In summary, misdiagnosis and delayed treatment are often caused by non-specific clinical manifestations, complex complications, and neglect of epidemiological history. Detailed history, proper interpretation of laboratory results, and knowledge of rare complications of human brucellosis facilitate early diagnosis of the disease.

## Data availability statement

The raw data supporting the conclusions of this article will be made available by the authors, without undue reservation.

## Ethics statement

The studies involving humans were approved by the Ethics Committee of Changchun Infectious Disease Hospital and the Ethics Committee of the 1st Hospital of Jilin University, China. The studies were conducted in accordance with the local legislation and institutional requirements. Written informed consent for participation was not required from the participants or the participants’ legal guardians/next of kin in accordance with the national legislation and institutional requirements. Written informed consent was obtained from the individual(s) for the publication of any potentially identifiable images or data included in this article. Written informed consent was obtained from the participant/patient(s) for the publication of this case report.

## Author contributions

Conceptualization: YW; validation: YW and LS; writing—original draft preparation: LS and SW; writing—review and editing: YW; visualization: XiaL and XinL; supervision: YW; project administration: YW. All authors agree to be accountable for the content of the work. All authors contributed to the article and approved the submitted version.

## References

[B1] Al Jindan R. (2021). Scenario of pathogenesis and socioeconomic burden of human brucellosis in Saudi Arabia. Saudi J. Biol. Sci. 28, 272–279. doi: 10.1016/j.sjbs.2020.09.059 33424306 PMC7783673

[B2] Alkan S. Guclu-Kayta S. B. Vurucu S. Akca A. Yuksel C. Onder T.. (2022). A case of brucellosis presenting with infarction in the spleen. Klimik Dergisi/Klimik J. 35, 109–110. doi: 10.36519/kd.2022.3868

[B3] Alyousef M. Enani M. Elkhatim M. (2015). Acute brucellosis with splenic infarcts: A case report from a tertiary care hospital in Saudi Arabia. Case Rep. Infect. Dis. 2015, 940537. doi: 10.1155/2015/940537 26246924 PMC4515537

[B4] Antopolsky M. Hiller N. Salameh S. Goldshtein B. Stalnikowicz R. (2009). Splenic infarction: 10 years of experience. Am. J. Emerg. Med. 27, 262–265. doi: 10.1016/j.ajem.2008.02.014 19328367

[B5] D'anastasio R. Staniscia T. Milia M. L. Manzoli L. Capasso L. (2011). Origin, evolution and paleoepidemiology of brucellosis. Epidemiol. Infect. 139, 149–156. doi: 10.1017/S095026881000097X 20447329

[B6] de Figueiredo P. Ficht T. A. Rice-Ficht A. Rossetti C. A. Adams L. G. (2015). Pathogenesis and immunobiology of brucellosis: review of Brucella-host interactions. Am. J. Pathol. 185, 1505–1517. doi: 10.1016/j.ajpath.2015.03.003 25892682 PMC4450313

[B7] Dursun Z. B. Demiraslan H. Çelik İ. (2015). A case of splenic infarction associated with brucellosis which resolved with antimicrobial treatment. J. Microbiol. Infect. Dis. 2, 168–170. doi: 10.5799/jmid.123133

[B8] Edathodu J. Alamri M. Alshangiti K. A. Alfagyh N. S. Alnaghmush A. S. Albaiz F.. (2021). Clinical manifestations and treatment outcomes of human brucellosis at a tertiary care center in Saudi Arabia. Ann. Saudi Med. 41, 109–114. doi: 10.5144/0256-4947.2021.109 33818142 PMC8020648

[B9] Gwida M. Al Dahouk S. Melzer F. Rösler U. Neubauer H. Tomaso H. (2010). Brucellosis - regionally emerging zoonotic disease? Croat Med. J. 51, 289–295. doi: 10.3325/cmj.2010.51.289 20718081 PMC2931433

[B10] Hachfi W. Bellazreg F. Atig A. Ben Lasfar N. Kaabia N. Letaief A. (2012). Splenic infarction associated with acute brucellosis: a case report. Adv. Infect. Dis. 02, 89–91. doi: 10.4236/aid.2012.24014

[B11] Im J. H. Chung M. H. Lee H. J. Kwon H. Y. Baek J. H. Jang J. H.. (2020). Splenic infarction and infectious diseases in Korea. BMC Infect. Dis. 20, 915. doi: 10.1186/s12879-020-05645-9 33267828 PMC7708890

[B12] Kose S. Serin Senger S. Akkoclu G. Kuzucu L. Ulu Y. Ersan G.. (2014). Clinical manifestations, complications, and treatment of brucellosis: evaluation of 72 cases. Turk J. Med. Sci. 44, 220–223. doi: 10.3906/sag-1112-34 25536728

[B13] Lee J. H. Lee Y. M. Lee C. H. Choi C. S. Kim T. H. (2010). Splenic infarction associated with brucellosis in a non-endemic area. Infect. Chemother. 42, 48–50. doi: 10.3947/ic.2010.42.1.48

[B14] Odeh M. Pick N. Oliven A. Odeh M. (2000). Deep venous thrombosis associated with acute brucellosis: A case report. Angiology 51, 253–256. doi: 10.1177/000331970005100310 10744014

[B15] Orduña A. Almaraz A. Prado A. Gutierrez M. P. Garcia-Pascual A. Dueñas A.. (2000). Evaluation of an immunocapture-agglutination test (Brucellacapt) for serodiagnosis of human brucellosis. J. Clin. Microbiol. 38, 4000–4005. doi: 10.1128/JCM.38.11.4000-4005.2000 11060059 PMC87532

[B16] Saad M. A. Ahmed E. S. Alghamdi F. A. Fahmy Y. R. Amin Y. E. Saad A. A. (2021). Acute brucellosis associated with isolated splenic and left gastric artery vasculitis and acute ischemic bowel infarction. A systematic review of the most recent cases. Infez Med. 29, 469–474. doi: 10.53854/liim-2903-19 35146353 PMC8805487

[B17] Salgado F. Grana M. Ferrer V. Lara A. Fuentes T. (2002). Splenic infarction associated with acute Brucella mellitensis infection. Eur. J. Clin. Microbiol. Infect. Dis. 21, 63–64. doi: 10.1007/s10096-001-0655-3 11913506

[B18] Suvak B. Dulger A. C. Karadas S. Gonullu H. Bayram Y. Gonullu E.. (2016). Brucellosis-related acute pancreatitis: a rare complication of a universal disease. J. Int. Med. Res. 44, 131–135. doi: 10.1177/0300060515583078 26647073 PMC5536568

[B19] Tao Z. Chen Q. Chen Y. Li Y. Mu D. Yang H.. (2021). Epidemiological characteristics of human brucellosis - China 2016-2019. China CDC Wkly 3, 114–119. doi: 10.46234/ccdcw2021.030 34595016 PMC8393115

[B20] Uçmak F. Uçmak D. Beştaş R. Anli R. A. Adanir H. (2014). Acute brucellosis associated with leukocytoclastic vasculitis and splenic infarct. Infez Med. 22, 326–330. 25551851

[B21] Wang M. Zhu Q. Yang Q. Li W. Wang X. Liu W.. (2017). Intestinal brucellosis associated with celiac artery and superior mesenteric artery stenosis and with ileum mucosa and submucosa thickening. Medicine 96 (2), e5893. doi: 10.1097/MD.0000000000005893 28079834 PMC5266196

[B22] Yagupsky P. Morata P. Colmenero J. D. (2019). Laboratory diagnosis of human brucellosis. Clin. Microbiol. Rev. 33. doi: 10.1128/CMR.00073-19 PMC6860005 31722888

